# On the Causes of Plague

**Published:** 1845-11

**Authors:** 


*On the Causes of Plague.* By M. Hamont.—M. Hamont terminated his long memoir on the plague, the reading of which occupied more than one meeting. From the numerous facts which he brings forward, he concludes that plague owes its origin to a reunion of many different causes of insalubrity in the houses of the fellahs or peasants in Egypt, aided by heat, humidity, the unwholesome south wind, &c. He consequently concludes that plague is in a great measure the work of man, and not the result of an assumed epidemic or endemic constitution of the atmosphere. As experience, however, has proved that plague once originated may be propagated from man to man by contagion, or through means of goods, M. Hamont concludes, that lazarettoes are necessary in a sanitary point of view. He thinks, however, that the system of quarantine may be advantageously modified,—as, for instance, that vessels with clean bills of health should not be subjected to a longer quarantine than fifteen days, counting the days they have been on the voyage from the suspected port.—*Ibid, from Bul. de l'Acad. Roy. de Med.*

